# Mast Cells Are Not Essential for Pubertal Mammary Gland Branching

**DOI:** 10.1002/eji.70036

**Published:** 2025-08-17

**Authors:** Simran Kapoor, Clara M. Munz, Jimmy Marsden, Cyril Carvalho, Holly Tinsley, Marlene Magalhaes Pinto, Bert Malengier‐Devlies, Solvig Becker, Guillaume Seuzaret, Katelyn Patatsos, Ramazan Akyol, Amy B. Pederson, Gillian Wilson, Marc Dalod, Rebecca Gentek

**Affiliations:** ^1^ Institute For Regeneration and Repair Centre For Reproductive Health Centre For Inflammation Research University of Edinburgh Edinburgh UK; ^2^ Institute of Medical Sciences University of Aberdeen UK & Research Institute for Environmental and Occupational Health Université De Rennes I Rennes France; ^3^ Murdoch Children's Research Institute Royal Children's Hospital Victoria Australia; ^4^ Aix‐Marseille University CNRS INSERM Centre d'immunologie De Marseille‐Luminy (CIML) Marseille France; ^5^ Institute of Ecology and Evolution School of Biological Sciences University of Edinburgh Edinburgh UK; ^6^ Present address: Institute of Infection Immunity and Inflammation University of Glasgow Glasgow UK

**Keywords:** branching, mammary gland, mast cells, puberty

## Abstract

Mast cells are long‐lived, tissue‐resident immune cells of the myeloid lineage with cardinal functions in allergy and atopic disease. They are now increasingly recognized also for protective roles, for example against infections and venoms. Other functions originally assigned to mast cells in development and physiology, however, have been refuted, and for yet others, the true contribution of mast cells remains uncertain. Mast cells have been implicated in promoting ductal branching in the pubertal mammary gland, the organ that produces and secretes milk in mammals, but these findings are based on mouse models that are not mast cell‐specific. In this study, we therefore re‐addressed the impact of mast cells on mammary gland branching using several complementary genetic models, including a newly generated transgenic mouse line (*Ms4a2* ^lsl‐hDTR^). We report that neither constitutive deficiency of mast cells, nor their conditional ablation induced at puberty affects mammary gland branching. Our results thus dispute that mast cells promote this process in mice, at least in a unique and non‐redundant manner. This study adds to a growing body of work clarifying the biological roles of mast cells and further expands the toolbox available to the field of mast cell research.

## Graphical abstract

Mast cells colonise tissues during development and may be involved in maturation processes. In the mammary gland, they are thought to promote pubertal branching morphogenesis. Using new Kit‐independent models that are more mast cell‐specific, we demonstrate that neither constitutive deficiency nor induced depletion at puberty impairs mammary gland branching. Mast cells are thus not essential for this particular developmental process.

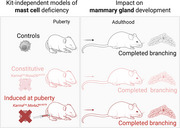

**Abbreviations**
TEBs: terminal end buds
TEDs: terminal end ducts

## 1 Introduction

The mammary gland is an organ specific to the mammalian reproductive system, and its primary function is milk production and secretion. It consists of milk ducts that spread throughout an adipose tissue pad and end in terminal end ducts (TEDs) and terminal end buds (TEBs), which facilitate duct branching and elongation during development and pregnancy. The mammary gland is also rich in resident immune cells and is indeed also of immunological importance: transfer of immunoglobulin antibodies and immune cells through the milk can support offspring immunity [1], and the gland also provides an immunological barrier during suckling.

The mammary gland develops in a series of discrete steps ranging from embryonic to postnatal stages. In mice, mammary gland development is initiated at day 10.5–11 of embryonic development (E10.5–E11) with the formation of ectodermal disks that, from E13.5 onwards, invade the underlying mesenchyme and eventually form the nipples. The developing ductal epithelium then starts branching, resulting in a few rudimentary branches present at birth [2, 3, 4, 5, 6]. These branches then remain largely quiescent and only grow allometrically until puberty, which marks the key period of mammary gland development. The start of ductal branching is often the first defining sign of puberty onset, and in humans is a developmental window of susceptibility to breast cancer [7, 8]. During puberty, the previously rudimentary ducts grow and TEBs are formed. Cells at the end of the TEBs then proliferate to invade the fat pad, and branches form through the bifurcation of TEBs [2]. This process repeats until the ductal tree has invaded the entire fat pad. Throughout the reproductive cycle, the mammary gland undergoes further dynamic changes, enabling it to fulfil its physiological functions. With pregnancy, the secretory epithelium forms alveoli to prepare for lactation. Upon weaning, these alveoli undergo involution, and the gland returns to a state similar to that before pregnancy. Mammary gland remodeling is a carefully orchestrated process that relies on internal and external signals. Growth factors and hormones act on the mammary epithelium and stroma to facilitate branching and alveoli formation. Whilst fetal mammary gland development is independent of estrogen [9], it is suppressed by androgens in male mice. Testosterone exposure results in regression of mammary gland development [10], and androgens cause mesenchymal cells to condense around the developing epithelium, preventing further gland development [11]. On the contrary, pubertal branching depends on estrogen receptor α expression in epithelial cells [9]. During puberty, progesterone acts on stromal cells to promote branching [3, 12]. Finally, remodeling during pregnancy is induced by prolactin, which acts together with progesterone to promote alveolar growth. Local influences from stromal cells further contribute to remodeling. For example, fibroblasts in the mammary gland stroma communicate with the developing epithelium [13] by providing an extracellular matrix capable of maintaining the mammary glands [14].

Another player in the morphogenesis of mammary gland ducts is immune cells: alveolarization during pregnancy is impaired in mice deficient in IL‐4 and IL‐13 [15], and pubertal duct elongation and branching are reduced in mice lacking eosinophils and macrophages in the mammary gland [16, 17, 18, 19]. This also seems to be true for mice deficient in mast cells, which show a reduction in the number of TEBs, as well as numbers and length of ducts during puberty [20]. Mast cells are potent myeloid effector cells best known as mediators of allergy and anaphylaxis and for their implications in atopic disease. They also have protective roles, for example, against bacteria and venoms [21, 22, 23] and by promoting avoidance behavior toward food allergens [24, 25]. However, their contribution to normal tissue functioning and development remains heavily debated [26]. This is at least in part due to limitations in the tools used to study mast cells. Their maturation and survival depend on stem cell factor, and inactivating mutations in its receptor Kit cause mast cell deficiencies. Traditionally, Kit‐dependent mouse models have therefore been used to investigate mast cell functions [27]. The usefulness of these models is limited, however, because Kit mutations also affect other immune and nonimmune cells, as evidenced by several defects in Kit‐mutant mice that are unrelated to mast cells [27]. This is of particular importance to the mammary gland, since Kit is expressed by the breast epithelium [28, 29]. Mast cells can be found near the ducts at the onset of puberty, and it has been suggested that they can directly interact with the ductal epithelium [20]. Pubertal branching is stunted in the absence of mast cells, a defect that is rescued by adulthood through an unknown compensatory mechanism [20]. However, these findings were made in mutant *Kit* ^Wsh^ mice. It is currently unknown if mice with Kit‐independent mast cell deficiency copy this phenotype, and hence, whether this defect is indeed attributable to the absence of mast cells.

In this study, we re‐addressed the involvement of mast cells in pubertal mammary gland branching using Kit‐independent genetic mouse models of mast cell deficiency, including a new Cre‐responsive conditional transgenic line in which mast cell depletion can be induced by administration of Diphtheria toxin. Unexpectedly, we found no defects in mammary gland branching in the absence of mast cells. Our data therefore dispute a critical contribution of mast cells to this developmental process, at least in a unique and non‐redundant manner.

## 2 Results

### 2.1 Mast Cells Are Present in Postnatal Mammary Glands and Functional at Puberty

**FIGURE 1:**
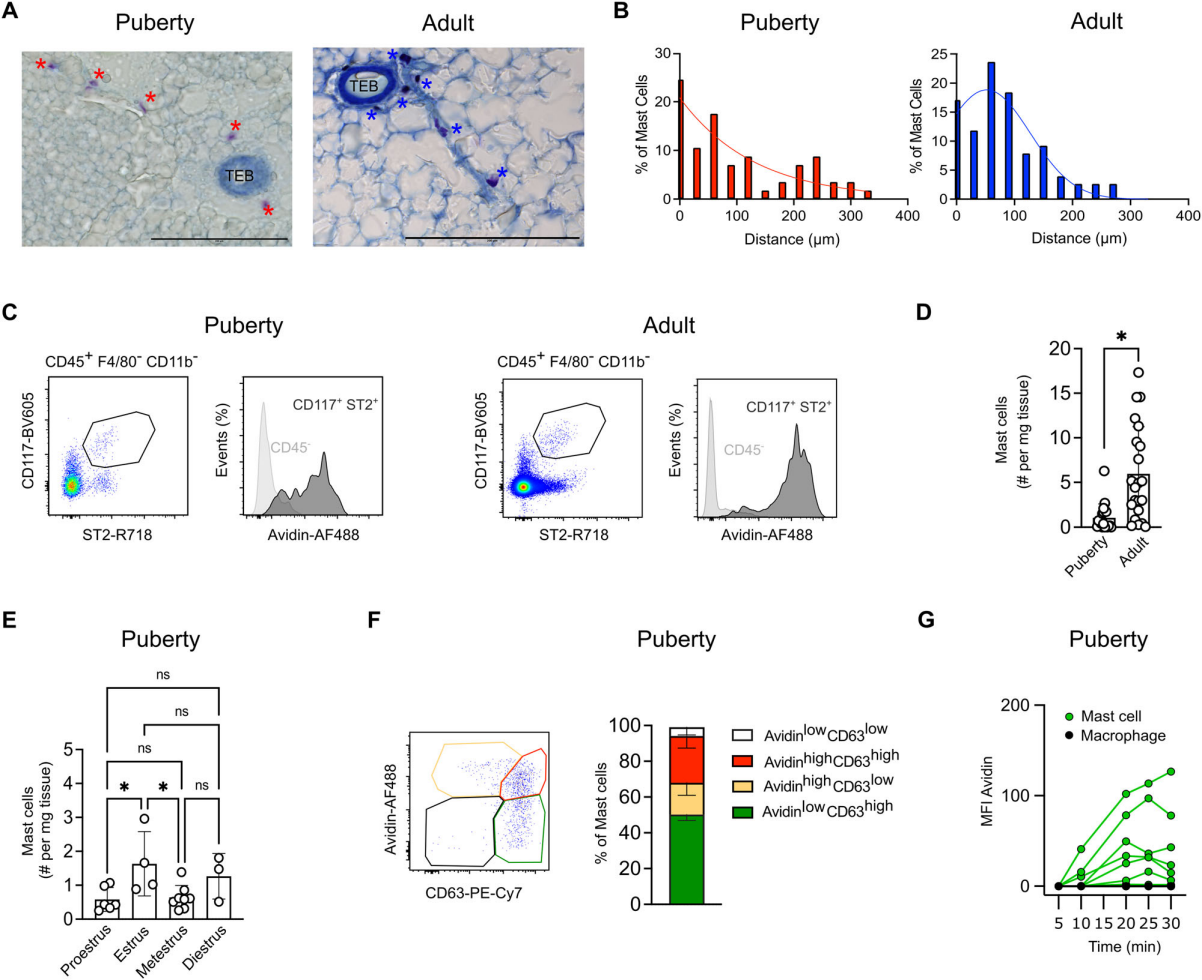
Mast cells are present in the postnatal mammary gland and are activated at puberty. (A, B) Histological analysis of mast cells in the mouse mammary gland. Pubertal (left) and adult (right) mammary glands were stained with Toluidine Blue, a metachromatic dye for staining mast cell granules (purple). (A) Representative images. Scale bar = 200µm. (B) Mast cell distribution. The frequency of mast cells within the indicated distances (in micrometers) from terminal end buds (TEBs) was quantified. Data are representative (A) and cumulative (B) of four individual mice per developmental time point. (C) Flow cytometry identification strategy for mast cells within the pubertal (5.5 weeks; left panels) and adult (8–15 weeks; right panels) mammary gland. (D) Quantification of mast cells (CD117 (Kit^+^) Avidin^+^) in pubertal and adult mammary glands. Data in (C, D) are from at least 19 mice per age. Quantified data are shown as mean with error bars indicating the SD. *p < 0.05 as determined by the Mann–Whitney test. ns = not significant. (E) Mast cell numbers (CD117^+^ (Kit^+^) Avidin^+^) in pubertal mammary glands stratified by estrus stage. Data from at least three mice per estrus stage. Quantified data are shown as mean with error bars indicating the SD. *p < 0.05 as determined by one‐way ANOVA test. ns = not significant. (F) Flow cytometric assessment of primary mammary gland mast cell activation states at puberty (5.5 weeks). Mast cells were gated as CD117^+^ (Kit^+^) ST2^+^, as shown in (C). The levels of degranulation were estimated by intracellular Avidin staining for heparin‐containing granules and surface staining of CD63. Data are representative of (left) and quantified (right) for six individual mice. (G) Time course of mast cell degranulation upon stimulation with Substance P. Mast cells (green) were sorted from the pubertal (5.5 weeks) mammary gland and subjected to short‐term culture and longitudinal imaging for release of heparin‐containing granules. Macrophages (black) isolated from the same glands were analyzed as negative controls.

In principle, mast cells could regulate ductal branching via locally restricted mechanisms or through longer‐range signaling, since granules can travel far upon release. The mammary gland consists of ductal epithelium and stromal cells, including adipocytes. Mast cells can be found associated with all these structures, that is, near TEBs and embedded in the adipose stroma (Figure 1A). Mast cells can also be detected close to the lymph node and major blood vessels (Figure S1A), as previously reported [20]. However, in both pubertal and adult glands, more than half (54% at puberty and 59% in adults) of mast cells are located within 100µm of TEBs (Figure 1B).

Like other immune cells, mast cells are commonly studied by flow cytometry, and they can also be identified in single‐cell transcriptomics datasets of samples obtained by tissue dissociation [30]. Nonetheless, to our knowledge, this approach has not been exhaustively applied to mast cells in the mammary gland. Therefore, we initially characterized mast cells in mammary glands from pubertal (5.5 weeks) and adult (aged 8–15 weeks) virgin mice by flow cytometry. Mast cells were identified as hematopoietic (CD45^+^) cells that lack markers for other myeloid lineages (CD64^−^ and/or F4/80^−^, CD11b^low^) and express CD117 (Kit^+^) and the IL33‐receptor alpha (ST2^+^) (Figure 1C; Figure S1B; Note: a similar gating strategy was also used for peritoneal cavity as a reference tissue [Figure S1C]). A characteristic feature of mast cells is their intracellular granules. These are densely packed with potent effectors and differ in their composition between distinct classes of mast cells. Mammary gland mast cells are of the connective tissue type [20, 30]. These types of mast cells feature heparin‐containing intracellular granules, which can be stained with Avidin [31, 32, 33]. Indeed, most CD117^+^ (Kit^+^) ST2^+^ mast cells also stained positive with fluorescently labelled Avidin in the mammary gland at puberty and in adulthood (Figure 1C). We thus used this as the primary gating strategy to identify mammary gland mast cells throughout this study. Compared with puberty, mast cells were approximately sixfold more abundant in adult glands (Figure 1D). We also noted variation between individual mice. This was true both at puberty and in adult glands. In other organs of the reproductive system, like the uterus, mast cell numbers vary with the estrus cycle [34, 35]. Indeed, in rats, mast cells are more abundant at diestrus [36]. Moreover, histamine levels are higher in the murine gland at estrus [37, 38]. We therefore stratified pubertal animals by estrus stage. This showed that mast cell numbers are slightly elevated at estrus, but comparable otherwise (Figure 1E). Of note, we also obtained significantly fewer mast cells from abdominal fat pads of adult male mice compared with females (Figure S1D). Whilst sex‐specific differences in mast cell numbers may exist in the adipose stroma, this could indicate that most of the mast cells we identify in females by flow cytometry are duct‐associated, in keeping with our imaging analysis.

Since mast cells are thought to promote ductal branching during puberty, we next wanted to assess the activation status of the mast cells present at this stage. Mast cell activation results in granule release, which can be measured by surface staining for the tetraspanin CD63, a component of granule membranes that becomes externalized upon degranulation [39]. To gauge if mast cells can be activated in the pubertal gland, we thus determined surface expression of CD63 alongside levels of intracellular heparin‐containing granules on freshly isolated cells *ex vivo*, without experimental treatments to trigger degranulation (Figure 1F). Using this assay, most mast cells are either actively degranulating (Avidin^high^ CD63^high^, on average 26.2%) or have recently been activated (Avidin^low^ CD63^high^, 50.2%), whilst only minor fractions appear to be in a non‐active (Avidin^high^ CD63^low^, 17.8%) or immature or refractory stage (Avidin^low^ CD63^low^, 5%), indicating that at puberty, mammary gland mast cells can degranulate. To further confirm their functionality, we performed an assay to investigate whether mast cells sorted from the pubertal mammary gland are able to degranulate upon stimulation. In this assay, we determined the extent of degranulation by measuring heparin‐containing granules (identified by Avidin staining) on the cell surface released upon stimulation with Substance P. Mast cells from the mammary gland were able to degranulate upon stimulation, indicated by increasing granule presence over the course of 30 min (Figure 1G; Figure S1E). As expected, macrophages sorted from the same glands could not be stimulated in this manner.

In summary, we confirmed that mast cells are present in the adult and pubertal mammary gland. Moreover, we provide evidence that mast cells are also functional in the pubertal gland.

### 2.2 Mammary Glands of *Karma* ^Cre^:Rosa26^lsl‐DTA^ Mice Are Constitutively Deficient in Mast Cells

**FIGURE 2:**
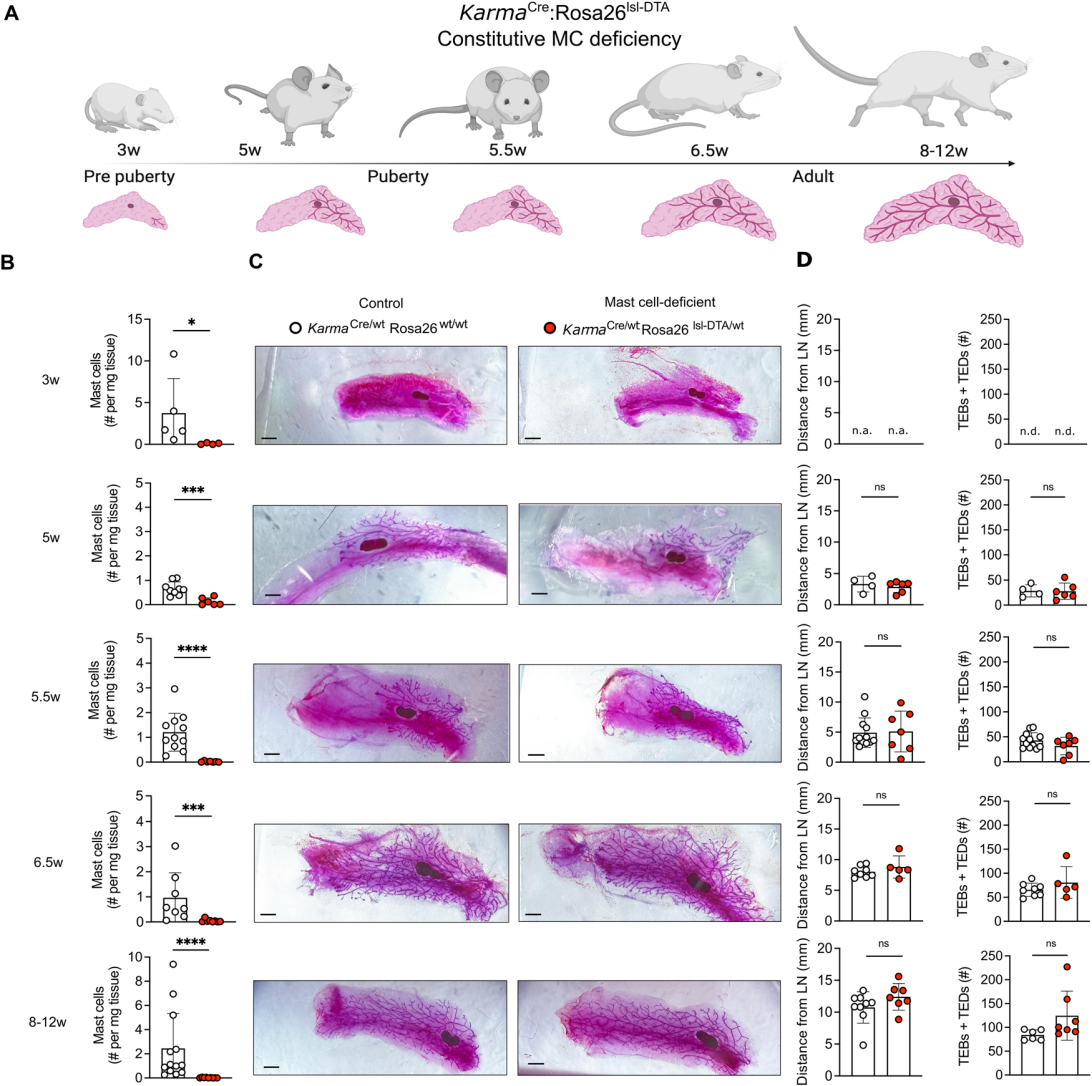
**Constitutive mast cell deficiency has no impact on mammary gland branching. (A)** Schematic illustrating experimental approach. *Karma* ^Cre/wt^:Rosa26^lsl‐DTA^ mice were analyzed at 3, 5, 5.5, 6.5, and 8–12 weeks (w) of age. **(B)** Time course analysis showing mast cell numbers in *Karma* ^Cre/wt^: Rosa26^lsl‐DTA/wt^ mice (red) compared with mast cell‐proficient littermate controls (*Karma* ^Cre/wt^:Rosa26^wt/wt^ mice; white). Mast cells were quantified by flow cytometry at the indicated developmental time points. **(C)** Representative mammary glands of mast cell‐deficient mice (*Karma* ^Cre/wt^:Rosa26^lsl‐DTA/wt^, right) and mast cell‐proficient littermates (*Karma* ^Cre/wt^:Rosa26^wt/wt^; left), analyzed for branching by Carmine staining at the indicated life stages. Scale bars = 1mm. **(D)** Analysis of postnatal mammary gland branching on Carmine‐stained mammary glands of mast cell‐deficient mice (*Karma* ^Cre/wt^:Rosa26^lsl‐DTA/wt^; red) and mast cell‐proficient littermates (*Karma* ^Cre/wt^:Rosa26^wt/wt^; white) across postnatal development. The distance of the branching front and the number of terminal end buds (TEBs) and end ducts (TEDs) past the middle of the lymph node were measured. Data are representative (**C**) and cumulative (**B, D**) of at least five individual mice per group from at least two independent experiments. Data in (**B, D**) are shown as mean with error bars indicating the SD. ****p < 0.0001, ***p < 0.001, *p < 0.05 as determined by Mann–Whitney test. ns = not significant.

The data implicating mast cells in ductal branching in the mammary gland are primarily based on *Kit* ^Wsh^ mice [20]. To investigate if the transient delay in branching observed in these mice is indeed attributable to mast cells, we sought to use mice in which mast cell deficiency does not depend on mutations in *Kit*. First, we wanted to utilize the Cre loxP system to constitutively ablate mast cells. The *Karma* gene (also known as *Gpr141b*) encodes an orphan G‐protein‐coupled receptor. In *Karma* ^Cre^:Rosa26^lsl‐DTA^ mice, Cre activity results in the removal of a stop cassette that otherwise prevents expression of the Diphtheria toxin alpha subunit (DTA), thereby inducing death specifically in cells expressing *Karma*, the gene driving Cre recombinase [40]. *Karma* ^Cre^ targets connective tissue mast cells in the skin [40], and *Karma* ^Cre/wt^:Rosa26^lsl‐DTA/wt^ mice are profoundly deficient in peritoneal cavity mast cells (Figure S2A). We thus assessed the level of mast cell depletion in the postnatal mammary gland of *Karma* ^Cre^:Rosa26^lsl‐DTA^ mice (Figure 2A). Considering our focus on pubertal maturation, we analyzed the window around puberty with higher granularity. Encouragingly, *Karma* ^Cre^:Rosa26^lsl‐DTA^‐mediated virtually complete ablation of mast cells in the mammary gland at all stages analyzed, that is, prior to puberty (3 weeks), at the onset (5 weeks), during (5.5 weeks), and at conclusion (6.5 weeks) of puberty, as well as in mature adults (8–12 weeks) (Figure 2B).

### 2.3 Constitutive Mast Cell Deficiency in *Karma* ^Cre^:Rosa26^lsl‐DTA^ Mice Has no Effect on Mammary Gland Branching

Having established *Karma* ^Cre^:Rosa26^lsl‐DTA^ mice as a tool for robust mast cell deficiency in mammary glands, we next performed Carmine staining to investigate if these mice show any branching defects. If mast cells did indeed promote pubertal branching, then these mice should exhibit impaired ductal branching like previously reported in *Kit* ^Wsh^ mice [20]. Because any defect might be transient in nature and compensated by adulthood, as is the case in *Kit* ^Wsh^ mice [20], we performed a time course analysis. Prior to puberty, at 3 weeks of age, the mammary glands only contain rudimentary ducts. These were indistinguishable in mast cell‐deficient *Karma* ^Cre/wt^:Rosa26^lsl‐DTA/wt^ and *Karma* ^Cre/wt^:Rosa26^wt/wt^ control mice (Figure 2C,D). In our hands, the onset of puberty occurs at around 5 weeks of age, as evidenced by ductal outgrowth beyond the lymph node and the first signs of branching. To our surprise, despite the absence of mast cells, *Karma* ^Cre/wt^:Rosa26^lsl‐DTA/wt^ mice did not show defects in branching at any stage of postnatal development (Figure 2C,D). Between 5.5 and 6.5 weeks of age, ducts spread out and branches multiply, and the process is fully concluded by 8–12 weeks, when the entire fat pad is covered in ducts. However, at neither of these time points did we observe differences between mast cell‐deficient *Karma* ^Cre/wt^:Rosa26^lsl‐DTA/wt^ animals and mast cell‐proficient controls (*Karma* ^Cre/wt^:Rosa26^wt/wt^). This was true for the extent of branching, as determined by the distance of the branching front beyond the lymph node, as well as the number of TEBs and TEDs. These data suggest that pubertal mammary gland branching occurs independently of mast cells, unlike what is currently thought.

### 2.4 Specific Ablation of Mast Cells at the Onset of Puberty Has no Effect on Mammary Gland Branching in *Karma* ^Cre^:*Ms4a2* ^lsl‐hDTR^ Mice

A potential caveat of the *Karma* ^Cre^:Rosa26^lsl‐DTA^ model is that *Karma* ^Cre^ also targets dendritic cells, specifically conventional (c)DC1 [40]. DCs are present in the mammary gland, including subsets that resemble cDC1 [41, 42]. They have also been implicated in regulating ductal branching, albeit in an inhibitory manner. Indeed, it has been reported that CD11c^+^ cells, which encompass DCs and macrophages, inhibit branching via a mechanism that involves antigen presentation to T cells [43]. Moreover, cDC1 are reduced in *CD11c* ^Cre^:*Irf8* ^fl/fl^ mice, and they show a trend toward moderately increased branching at 6 weeks of age [44]. Whether these effects are due to targeting cDC1 has not been addressed. However, if branching was indeed promoted by mast cells and inhibited by cDC1s, then it is possible that simultaneous ablation of both has a net zero effect. Consequently, any effects of mast cell deficiency might be “masked” by concomitant absence of cDC1s. DCs are very sparse in the mammary gland at puberty [42] and, in our hands, not significantly depleted in pubertal and adult *Karma* ^Cre^:Rosa26^lsl‐DTA^ mice (Figure S2B). In addition, the abundance of other immune cell populations like macrophages reassuringly also seemed unaffected in pubertal and adult *Karma* ^Cre^:Rosa26^lsl‐DTA^ mice (Figure S2C). Nonetheless, we wanted to exclude possible effects of the deficiency of DCs and other lineages in the mammary gland on branching. To circumvent targeting DCs and other lineages, we therefore revised an alternative genetic strategy that exploits the efficacy of *Karma* ^Cre^ to enable selective ablation of mast cells, but not cDC1s or other lineages. To do so, we crossed *Karma* ^Cre^ mice to a new transgenic line that we generated here. The latter is a derivation of the *Ms4a2* ^hDTR^ model, also known as “Red mast cell and basophil” (RMB) mice [45] in which the human Diphtheria toxin receptor (hDTR) is expressed under control of the *Ms4a2* gene, which encodes the beta subunit of the IgE receptor. In these mice, mast cells and the closely related basophils are sensitive to Diphtheria toxin‐mediated ablation, because they both express *Ms4a2* [45]. Reassuringly, delivery of the toxin into the mammary fat pad of prepubertal *Ms4a2* ^hDTR/wt^ mice (Figure S3A) resulted in complete loss of mast cells in the mammary gland (Figure S3B). Despite local administration of Diphtheria toxin, we found that mast cells were also efficiently ablated in the peritoneal cavity (Figure S3C), indicating systemic action.

To further refine this approach, we rendered the *Ms4a2* ^hDTR^ system conditional by introducing a loxP‐flanked stop cassette in front of the sequence coding for hDTR (see Section 4). This approach restricts Diphtheria toxin‐sensitivity to cells that have undergone Cre‐mediated recombination and express *Ms4a2*. We refer to this new line here as *Ms4a2* ^lsl‐hDTR^. Intercrossing *Karma* ^Cre^ and *Ms4a2* ^lsl‐hDTR^ mice results in a combined model in which only mast cells are susceptible to depletion by Diphtheria toxin, but not cDC1s or basophils, since these respectively lack expression of *Ms4a2* or *Karma*.

**FIGURE 3:**
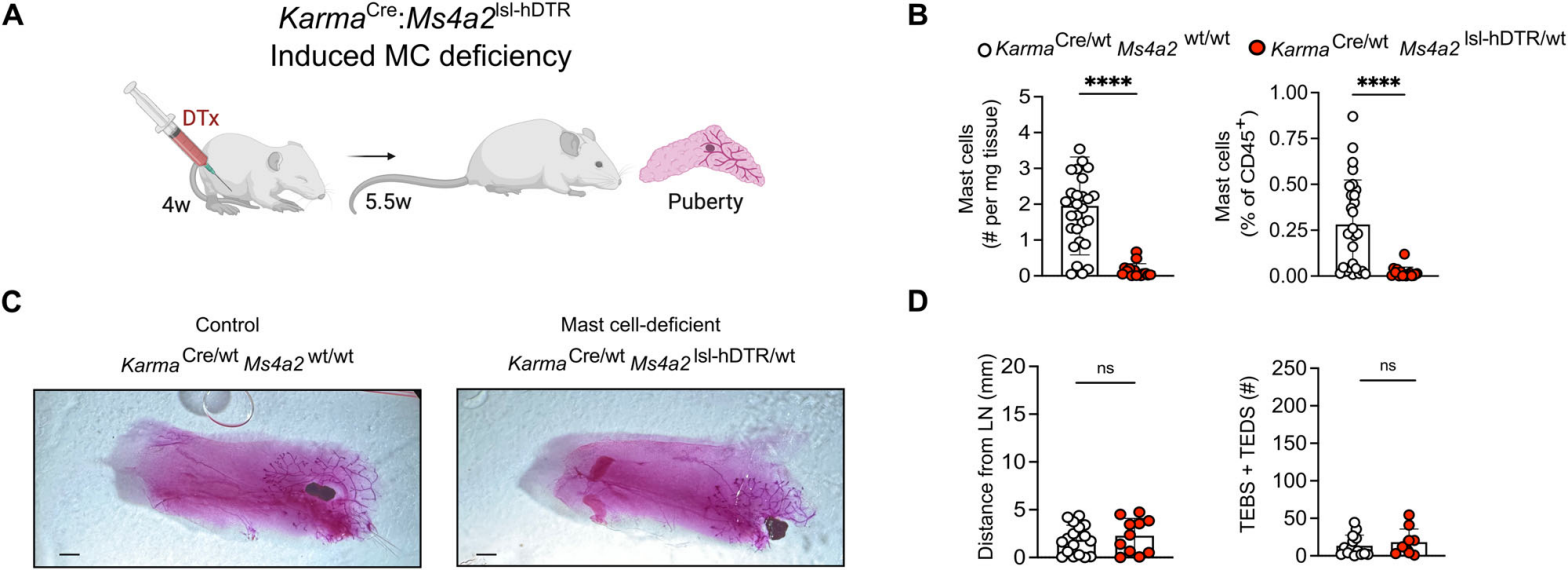
**Mast cell deficiency induced at puberty does not affect mammary gland branching. (A)** Diagram illustrating experimental approach. To induce mast cell ablation, *Karma* ^Cre/wt^:*Ms4a2* ^lsl‐hDTR/wt^ mice were treated with 1 µg Diphtheria toxin at 4 weeks (w) of age by subcutaneous injection into the mammary gland fat pad. Littermate control animals (*Karma* ^Cre/wt^:*Ms4a2* ^wt/wt^) were treated the same way. Animals were analyzed at puberty (5.5 weeks of age). **(B)** Mammary gland mast cell numbers and relative abundance within hematopoietic compartment (% within CD45^+^ cells) as determined by flow cytometry. **(C, D)** Branching as determined by Carmine staining of pubertal mammary glands from 5.5 weeks‐old mast cell‐deficient (*Karma* ^Cre/wt^:*Ms4a2* ^lsl‐hDTR/wt^; right/red) and mast cell‐proficient mice (*Karma* ^Cre/wt^:*Ms4a2* ^wt/wt^ mice; left/white). **(C)** Representative photographs of Carmine‐stained mammary glands. Scale bars = 1 mm. **(D)** The extent of branching was measured as the distance of branches and the number of terminal end buds (TEBs) and end ducts (TEDs) from the middle of the lymph node. Data are representative (**C**) and cumulative (**B, D**) of at least 10 individual mice per group from at least four independent experiments. Data in (**B, D**) are presented as mean with error bars indicating the SD. ****p < 0.0001 as determined by the Mann–Whitney test. ns = not significant.

Indeed, administration of a single dose of 1 µg Diphtheria toxin by subcutaneous injection into the mammary fat pad just prior to the onset of puberty at 4 weeks (Figure 3A; Figure S3D) specifically resulted in the absence of mast cells in the mid‐pubertal gland of *Karma* ^Cre^:*Ms4a2* ^lsl‐hDTR^ mice at 5.5 weeks (Figure 3B), but not other lineages including cDC1s and macrophages (Figure S3E,F). We chose this time point because in our hands, most branching occurred between 5.5 and 6.5 weeks (Figure 2C). We then used this experimental strategy to determine if conditional ablation of mammary gland mast cells at the onset of puberty affects ductal branching in this critical window. Like in the *Karma* ^Cre^:Rosa26^lsl‐DTA^ model, however, we found no difference in the extent of duct migration into the fat pad and branching between mast cell‐deficient (*Karma* ^Cre/wt^:*Ms4a2* ^wt/hDTR^) and control mammary glands (*Karma* ^Cre/wt^:*Ms4a2* ^wt/wt^) (Figure 3C,D). Moreover, branching was fully completed in adult (8–12 weeks) glands treated at 4 weeks (Figure S3G,H), similar to *Karma* ^Cre^:Rosa26^lsl‐DTA^ mice. However, unlike peritoneal mast cells that remained absent in adult animals given Diphtheria toxin at 4 weeks (Figure S3I), mast cell numbers were indistinguishable from control animals in adult mammary glands injected at the onset of puberty (Figure S3J).

Collectively, we obtained data in several complementary and *Kit*‐independent models that challenge the notion that mast cells promote pubertal branching in the mammary gland, at least in a unique and essential manner.

## 3 Discussion

In this study, we investigated whether mast cells promote ductal branching of the mammary gland during puberty, as previously reported in *Kit* ^Wsh^ mice [20]. Using two complementary alternative genetic models for either constitutive or selective inducible ablation of mast cells, we found no evidence for an involvement of mast cells in this developmental process.

There are several possible explanations for the absence of a branching phenotype, which are not mutually exclusive. First, as introduced, expression of Kit is not restricted to mast cells or even the immune system. This includes the epithelium of the mammary gland [28, 29]. Indeed, many roles previously assigned to mast cells in *Kit*mutant mice have been refuted in Kit‐independent models. For example, *Cpa3* ^Cre^ or “Cre master” mice lack mast cells due to Cre‐mediated cytotoxicity, and unlike *Kit*mutant mice, these are susceptible to antibody‐induced arthritis [46], autoimmune encephalomyelitis [46], and diet‐induced obesity [47]. The latter has also been confirmed in *Mcpt5* ^Cre^:Rosa26^lsl‐DTA^ mice [48]. In the mammary gland, Kit is expressed in epithelial cells, where it has been associated with growth and maintenance [28, 29, 49, 50]. Therefore, it is conceivable that direct effects on mammary epithelial cells, rather than mast cells, are at least partially responsible for the temporary branching defect reported in Kit‐mutant mice. Our findings thus add to a growing body of work showcasing the limitations of Kit‐based models for mast cell research, and for the first time, highlight these in a developmental context. Whilst the use of Kit‐mutant versus Kit‐independent mice could explain the discrepancy between our findings and the literature, we acknowledge that Lilla and Werb [20] also observed stunted branching at puberty in mice treated with chromolyn sodium to prevent mast cell degranulation, as well as in mice deficient in Dipeptidyl peptidase I (DPPI) [20]. Chromolyn sodium is commonly used as a mast cell stabilizing agent, but can also impact additional cell types, including other granulocytes, lymphocytes, and stromal cells like fibroblasts, endothelial, and epithelial cells. Whilst some of these may be indirect, direct effects of chromolyn sodium on other cell types have also been reported *in vitro* and *in vivo* [51, 52, 53]. Indeed, chromolyn can even suppress biological responses in the absence of mast cells [54], calling its use as a specific and efficient mast cell stabilizer into question. In a similar manner, DPPI is needed for the activation of mast cell proteases, notably chymases [55]. However, it also mediates activation of proteases in cytotoxic lymphocytes, monocytes, and neutrophils [56].

Second, although we found no branching defects in either *Karma* ^Cre^:Rosa26^lsl‐DTA^ or *Karma* ^Cre^:*Ms4a2* ^lsl‐hDTR^ mice, it remains possible that mast cells do contribute to the regulation of gland remodeling. However, rather than having unique, non‐redundant effects, they may normally do so in cooperation with other cell types. Alternatively, other cells may functionally compensate at least when (only) mast cells are absent, even if they physiologically are not the main drivers of the process. Such concerted action of mast cells with other cells has been described in the uterus. To facilitate increased blood flow to growing fetuses, uterine spiral arteries need to be transformed from thick‐walled vessels to vessels with enlarged lumens during gestation. Fetal growth restriction occurs if this process is insufficient. This occurs in *Kit* ^Wsh^ mice and can be rescued by reconstitution with wild‐type mast cells [34]. On the contrary, *Cpa3* ^Cre^ mice only show a mild spiral artery remodeling defect that does not cause fetal growth restriction [57]. Spiral artery remodeling, however, requires smooth muscle cells to become apoptotic, and this is mediated by Mcpt5 [58]. In the uterus, Mcpt5 is produced not only by mast cells, but also by a subset of natural killer (NK) cells [58]. Akin to *Cpa3* ^Cre^ mice that lack only mast cells but not NK cells, those lacking just NK cells also only show a mild defect in spiral artery remodeling [58]. Profoundly defective uterine vascular remodeling and fetal growth restriction are therefore only observed in mice with combined deficiency in both mast cells and NK cells, achieved either genetically in *Mcpt5* ^Cre^:Rosa26^lsl‐DTA^ mice or via antibody‐mediated depletion of NK cells in mast cell‐deficient *Cpa3* ^Cre^ mice [58]. Moreover, numbers of uterine mast cells are increased in IL‐15 knockout mice, which are genetically deficient in NK cells, and conversely, uterine NK cell numbers are higher in *Kit* ^Wsh^ mice compared with mast cell‐proficient controls [59]. This underscores that a compensatory increase in other immune cells can indeed occur in the absence of mast cells, and that this may mask defects. Given how vital spiral artery remodeling is to reproductive success, it is perhaps unsurprising that a complex regulatory system with several layers has evolved. In the mammary gland, such a scenario could explain why branching is unaffected in mice with selective mast cell deficiency, that is, *Karma* ^Cre^:*Ms4a2* ^lsl‐hDTR^ (in which mammary gland DCs are unaffected), but is delayed at puberty in DPPI knockout mice or following treatment with chromolyn sodium [20], which may also affect other cells, as discussed above. These considerations further underscore the complexity of choosing appropriate genetic models to study mast cell functions, even for Kit‐independent approaches. As the example of the uterus illustrates, mast cell depletion can have different outcomes depending on the (trans)gene used.

Finally, it is also possible that diverse types of mast cells exist within the mammary gland, which may have distinct biological roles. Indeed, it is increasingly recognized that mast cells are heterogenous beyond the classic dichotomy of connective‐ and mucosal‐type mast cells [30, 60]. Mast cells also dynamically change across life stages [30, 61, 62]. One aspect in which mast cells differ is their developmental origin. At mucosal sites, mast cells are predominantly derived from hematopoietic stem cells (HSCs) [30, 62, 63, 64]. In many connective tissues, however, mast cells are long‐lived and self‐maintained largely independently of HSCs in the bone marrow [30, 62, 63]. They also retain sizeable populations of the first mast cells that originate from yolk sac erythro‐myeloid progenitors [62, 63]. Mast cells from distinct sources also retain functional differences, at least upon immunological challenge, for example, by infections, or in dynamic settings like tissue remodeling during development [65]. Any such heterogeneity has not yet been resolved for mammary gland mast cells, but it is conceivable that they comprise subpopulations that, for example, are more or less responsive to hormones, and may thereby differently impact branching, perhaps even in opposing ways. Since *Karma* ^Cre^:Rosa26^lsl‐DTA^ and *Karma* ^Cre^:*Ms4a2* ^lsl‐hDTR^ mice lack all mast cells in the mammary gland, this is a possibility that we cannot account for here.

### 3.1 Data Limitations and Perspectives

One limitation is that we did not investigate other stages of mammary gland development. Mast cells colonize peripheral tissues in the fetus [62, 66, 67, 68, 69]. They might thus also already be present and functional in the fetal gland. We did not address this formally; however, the rudimentary, pre‐pubertal tree was indistinguishable between *Karma* ^Cre^:Rosa26^lsl‐DTA^ mice and mast cell‐proficient controls (Figure 2C). These mice are devoid of mammary mast cells at all stages tested, indicating that mast cells may also be dispensable for fetal gland morphogenesis. However, this would need to be investigated specifically at fetal stages. During pregnancy, ducts first undergo additional branching and later form milk‐secreting alveoli [3]. The number of mammary gland mast cells increases in mid to late pregnancy, coinciding with alveolar remodeling [70]. Furthermore, lactation involves the plasminogen cascade of serine proteases [71] and mast cell granules contain Plasma Kallikrein (PKa), an activator of the cascade [72]. Plasminogen and PKa are also needed for involution after weaning [71, 72]. Mast cells may thus be involved in alveolarization and involution. This could, in future work, be addressed in an inducible model to avoid potential confounding effects of maternal (uterine) mast cell deficiency. Intriguingly, in *Karma* ^Cre^:*Ms4a2* ^lsl‐hDTR^ mice, mammary gland (but not peritoneal) mast cells fully recovered by adulthood following depletion at puberty onset (Figure S3I,J), perhaps indicating requirements for mast cells in the postpubertal gland. *Ms4a2* ^lsl‐DTR^ will be a valuable tool to disentangle the developmental roles of mast cells in the gland and beyond.

## 4 Materials and Methods

### 4.1 Mouse Lines

Rosa26^lsl‐DTA^ (“DTA176”) mice were purchased from The Jackson Laboratory (strain #010527). *Karma* ^Cre^ mice [40] were gifted by Marc Dalod (Marseille, France), and the *Ms4a2* ^hDTR^ line (also known as “red mast cell and basophil” or RMB mice [45]) was obtained from Pierre Launay. *Ms4a2* ^lsl‐hDTR^ mice (official name: C57BL/6NRj‐*Ms4a2* ^2Ciphe^) are a new line we generated at the Centre d'immunophénomique (CIPHE) in Marseille (France). In these mice, the 3′‐UTR of the *Ms4a2* gene encoding the FcεRI β chain includes a cassette composed of an internal ribosomal entry site, a sequence coding for the fluorescent protein tdTomato (tdT), a 2A cleavage sequence, and the human diphtheria toxin receptor (hDTR). In this line, expression of the hDTR and tdT transgenes is prevented by a stop codon that is flanked by loxP Cre recognition sites. This stop codon is removed upon Cre‐mediated recombination, making these mice a Cre‐inducible version of the *Ms4a2* ^hDTR^ line. An overview of the transgene is shown in Figure S4.

### 4.2 Animal Ethics and Housing Conditions

All animal work was performed under project license (PPL) number PP1871024 in accordance with the Animals (Scientific Procedures) Act 1986 (ASPA). Mice were bred and housed at the animal facility in the University of Edinburgh (Edinburgh, UK) under specific pathogen‐free conditions at a controlled temperature of 22°C with a 12 h light/dark cycle. Access to food (irradiated chow pellets) and water (reverse osmosis water) was available ad libitum.

### 4.3 Diphtheria Toxin‐mediated Mast Cell Depletion

Diphtheria toxin (322326, Merck) was aliquoted in filtered PBS and stored at −80°C. *Ms4a2* ^hDTR^ and *Karma* ^Cre^:*Ms4a2* ^lsl‐hDTR^ mice were injected under general isoflurane anesthesia at 4 weeks of age with 1 µg Diphtheria toxin through the nipple into the fat pad of the fourth mammary gland.

### 4.4 Mast Cell Degranulation Assay

Mast cells were isolated from the mammary gland by flow cytometric cell sorting based on CD117 and ST2 expression. The sorted mast cells were then plated in Tyrode's buffer into μ‐Slide 18 Well plates (iBidi) with polymer‐coated coverslip bottom, precoated with ibiTreat (iBidi), and incubated for 2h at 37°C, 5% CO_2_. The Tyrode's buffer was supplemented with 2mg/mL Avidin (ThermoFisher) to allow for staining and detection of mast cell heparin granules upon degranulation. Under controlled atmospheric conditions (37°C) in a chamber, the mast cells were stimulated to degranulate with 100µM Substance P (Merck) or vehicle (Tyrode's buffer), and fluorescence was recorded 30 min poststimulation using the Zeiss Observer Live microscope system. Mean fluorescence intensity (MFI) was quantified for each individual mast cell (defined by phase contrast) using Fiji software.

### 4.5 Mammary Gland Harvest

The fourth abdominal mammary gland fat pads were removed at the indicated ages for analysis by flow cytometry and/or imaging. The lymph node was excluded from the gland collected for cell suspension preparation and flow cytometry, and glands intended for imaging were left intact.

### 4.6 Estrus Staging

Where assessed, estrus staging was performed by vaginal smear using x phosphate‐buffered saline (PBS) onto Superfrost Plus Adhesion Microscope Slides (Epredia). Vaginal smears were air‐dried and then fixed in ice‐cold 100% methanol, followed by staining with hematoxylin and eosin.

### 4.7 Peritoneal Lavage

Where done, peritoneal lavage was performed by injecting RPMI (supplemented with 2% FCS) into the abdominal cavity of mice immediately after cull, followed by gentle massaging to dislodge cells, and retrieval of the cell suspension using a syringe. The retrieved cell suspension was filtered through a 100µm filter membrane (John Stanair & Co) and stained for flow cytometry as detailed below.

### 4.8 Preparation of Single Cell Suspensions and Flow Cytometry

The dissected abdominal mammary gland fat pads were collected in RPMI supplemented with 2% FCS. Samples were cut manually into small pieces, followed by digestion in 0.8mg/mL Dispase II (Merck), 5mM CaCl_2_, 10mM HEPES (Merck), 0.1mg/mL DNaseI (Roche), 3mg/mL Collagenase D (Merck) in RPMI (supplemented with 2% FCS) at 37°C at 900 rpm in a Thermo‐Shaker (Grant‐bio) for 20min or until digested. Samples were filtered through a 100µm filter membrane (John Stanair & Co) and washed with FACS buffer (2% FCS, 2mM EDTA in PBS) to obtain a homogenous cell suspension. Cells were transferred to a 96‐well plate and incubated with 1:800 Zombie Live/Dead NIR (ThermoFisher Scientific) in PBS for 20–30min at room temperature in the dark. Cells were then washed with FACS buffer and centrifuged at 530*g* for 5min at 4°C. The supernatant was removed, and cells were incubated with 0.5% anti‐mouse CD16/32 Trustain Fx (Biolegend), 5% mouse serum (Invitrogen), and 5% rat serum (Merck) in FACS buffer for 20–30min at 4°C. Cells were then washed with FACS buffer and centrifuged at 530*g* for 5min at 4°C. Cells were then labelled with antibody mix diluted in FACS buffer and Brilliant Stain Buffer (BD) as detailed below for 20–30min at 4°C.

**Table:** 

Marker	Fluorophore	Supplier	Clone number	Dilution
CD45	BUV395	BD Biosciences	30‐F11	1:800
CD117 (Kit)	BV650	Biolegend	2B8	1:300
CD117 (Kit)	BV605	Biolegend	ACK2	1:300
CD11b	BV510	Biolegend	M1/70	1:400
CD64	BV711	Biolegend	X54‐5/71	1:300
F4/80	BV786	Biolegend	BM8	1:300
CD63	PE/Cyanine7	Biolegend	NVG‐2	1:200
ST2	APC	Biolegend	DIH9	1:200
ST2	R718	BD	U29‐93	1:200
MHCII	BV421	Biolegend	107632	1:200
CD11c	PerCP‐Cy5.5	Biolegend	117328	1:300
CD103	BUV805	BD	741948	1:300

Cells were then fixed in 4% paraformaldehyde solution (PFA) for 15min at room temperature away from light. Fixed cells were subsequently stained intracellularly with 1:10,000 Avidin‐AF488 (ThermoFisher Scientific) diluted in immunohistochemistry (IHC) buffer (0.1M Tris at pH 7.4, constituted with 0.5% bovine serum albumin, 2% Triton X‐100, and 0.05 g/mL milk powder) for 1 h at room temperature or overnight at 4°C in the dark. Cells were washed with FACS buffer and acquired in FACS buffer with counting beads (Invitrogen) (equivalent to 10,000 beads) added for quantification. Data was acquired using a 5L Fortessa (BD). Cells were pregated as live singlets. Mast cells were then defined as CD45^+^ CD11b^low^ F4/80^−^ and/or CD64^−^ CD117^+^ (Kit^+^) ST2^+^ and/or Avidin^+^. Dendritic cells were identified as CD45^+^ CD11b^−^ F4/80^−^ CD117^−^ (Kit^−^) Avidin^−^ CD11c^+^ MHCII^+^ CD103^+^. Macrophages were identified as CD45^+^ CD11b^+^ F4/80^+^.

### 4.9 Mammary Gland Imaging and Analysis

Dissected abdominal mammary gland fat pads were collected and spread onto Superfrost Plus Adhesion Microscope Slides (Epredia) and fixed for 24 h in Carnoy's buffer (75% ethanol, 25% glacial acetic acid).

To visualize mast cells within the mammary gland, fixed glands were first washed in PBS and then embedded in OCT matrix (CellPath). The tissue was then cryo‐sectioned at a thickness of 20 µm onto Superfrost Plus Adhesion Microscope Slides (Epredia). The sections were first air‐dried and then washed in PBS or distilled water to remove excess OCT. Each slide was stained for 2 min in Toluidine Blue (comprising 0.1% Toluidine blue O (Sigma) in 1% sodium chloride, pH 2.3–2.5) for metachromatic staining of mast cells, after which excess staining was removed by gently washing the slide in distilled water. The slides were mounted in aqueous Fluoromount G mounting media (ThermoFisher Scientific) and imaged. Images were acquired on the Zen Axioscan at 10× magnification and analyzed using Fiji software. A circular region of interest (ROI) with a fixed perimeter was drawn around two distinct TEBs of the gland. Two ROIs were used per gland to account for within‐gland variation, and overlapping regions were avoided when drawing up the ROIs. Mast cells within the ROIs were identified by metachromatic staining (characteristic purple shade) and marked using the multi‐point tool. The shortest distance of each mast cell from the TEB within the ROI was measured using the measure tool in Fiji, and the actual distance was calibrated using the appropriate scale in micrometers.

To visualise branching of the mammary gland, fixed glands were stained in Alum Carmine staining solution (0.1% carmine dye (ThermoFisher Scientific), 0.25% aluminium potassium sulfate dodecahydrate (ThermoFisher Scientific) in distilled water) for 2–7 days or until fully stained. Excess staining was removed by transferring the glands to a destaining solution (70% ethanol, 0.072% HCl) for 30min or until excess staining was sufficiently removed. Glands were then dehydrated in an alcohol series (75%, 80%, 95%, 100% ethanol) and finally cleared in xylene overnight or until the fat pad was cleared. The slides were mounted in Pertex mounting media (HistoLab) and imaged. Images were acquired on a Leica Stereomicroscope at 0.8x magnification and analyzed using Fiji software. Branching parameters were measured from a line perpendicular to the length of the fat pad bisecting the lymph node. The distance covered by branches (in mm) was measured from the lymph node to the longest branch terminating in a TEB. Numbers of TEBs and TEDs were counted in the direction of invading branching. Branching parameters were quantified by two independent parties blinded to experimental groups and/or genotypes.

### 4.10 Statistical Analysis

Unless otherwise stated, mean values and SD as specified in the respective figure legends were calculated in Prism (GraphPad), parametric (one‐way ANOVA, unpaired *t‐*tests) and nonparametric *t*‐tests (Mann–Whitney) were used where applicable. *p*‐values of less than 0.05 were considered statistically significant.

## Author Contributions

Study and experimental design: Simran Kapoor and Rebecca Gentek. Conduction, analysis, and/or interpretation of experiments and data: Simran Kapoor, Clara M. Munz, Jimmy Marsden, Cyril Carvalho, Holly Tinsley, Marlene Magalhaes Pinto, and Bert Malengier‐Devlies. Provision of samples or tools: Ramazan Akyol and Marc Dalod. Technical assistance: Solvig Becker, Guillaume Seuzaret, and Katelyn Patatsos. Intellectual contribution: Gillian Wilson and Amy B. Pederson. Drafting and revising the manuscript: Simran Kapoor, Rebecca Gentek, and Clara M. Munz. Funding acquisition: Rebecca Gentek and Simran Kapoor.

## Ethics Statement

All animal work was performed under project license (PPL) number PP1871024 in accordance with the Animals (Scientific Procedures) Act 1986 (ASPA).

## Conflicts of Interest

The authors declare no conflicts of interest.

## Peer Review

The peer review history for this article is available at https://publons.com/publon/10.1002/eji.70036.

## Supporting information




**Supporting file 1**: eji70036‐sup‐0001‐SuppMat.pdf

## Data Availability

All data related to this study are available either in the paper or in the Supporting Information Materials. Raw data are available upon request.
