# Supplementary material for: Mast Cells Are Not Essential for Pubertal Mammary Gland Branching
Source: Eur J Immunol. 2025 Aug 17;55(8):e70036. doi: 10.1002/eji.70036 (PMC12358710; doi:10.1002/eji.70036)
Supplement: Supplementary file 1 — Supporting file 1: eji70036‐sup‐0001‐SuppMat.pdf [file EJI-55-e70036-s001.pdf]

Figure S1

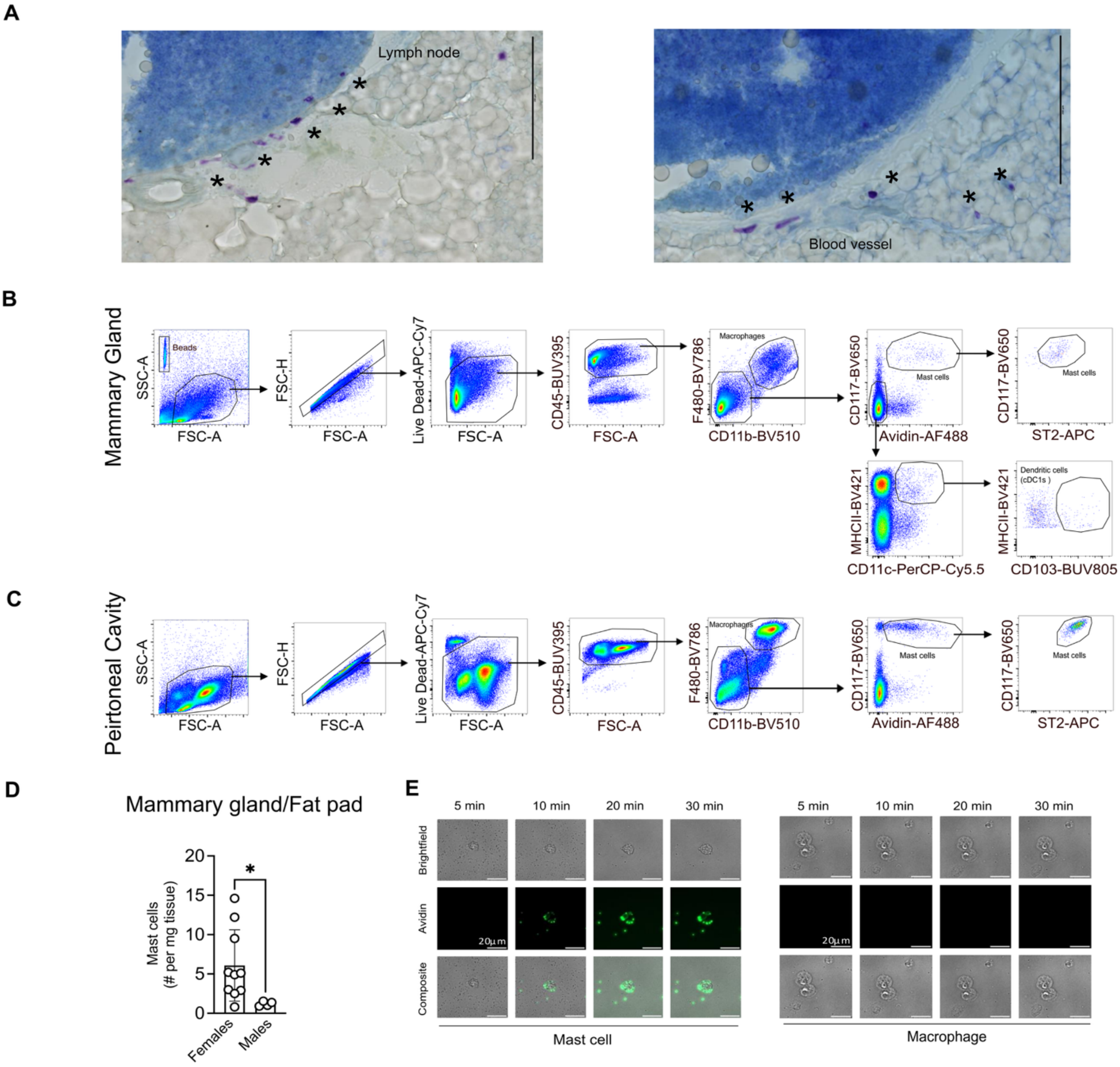

Figure S1 (related to Figure 1): Presence and functionality of mast cells in the mammary gland. (A) Representative images for localization of mast cells (purple) near the lymph node and blood vessels in the pubertal mammary gland stained by Toluidine Blue. Scale bar = 200µm. **(B)** Flow cytometry gating strategy used to identify mast cells (live single cells, $CD45^+$, $CD117^+$ (Kit^+) $Avidin^+$ $ST2^+$), macrophages (live single cells, $CD45^+$, $F4/80^+$ $CD11b^+$) and dendritic cells (live single cells, $CD45^+$, $CD117^-$ (Kit^-) $Avidin^-$, $CD11c^+$ $MHCII^+$ $CD103^+$) in mammary glands. **(C)** Flow cytometry gating strategy for identification of mast cells (live single cells, $CD45^+$, $CD117^+$ (Kit^+) $Avidin^+$ $ST2^+$) in the peritoneal cavity. **(D)** Flow cytometric mast cell quantification in the male abdominal fat pad compared to the female mammary gland at adulthood. Data are cumulative of at least 4 individual mice per group from at least 2 independent experiments. Data are shown as mean with error bars indicating the SD. *$p<0.05$ as determined by Mann-Whitney test. **(E)** Representative images for time course of degranulation assay. Mast cells sorted from the pubertal mammary glands (pooled from 4 individual mice) were stimulated with Substance P. Avidin staining (green) for heparin containing granules. As negative control, macrophages from the same pubertal mammary glands were analyzed the same way. Scale bar = 20mm.

Figure S2

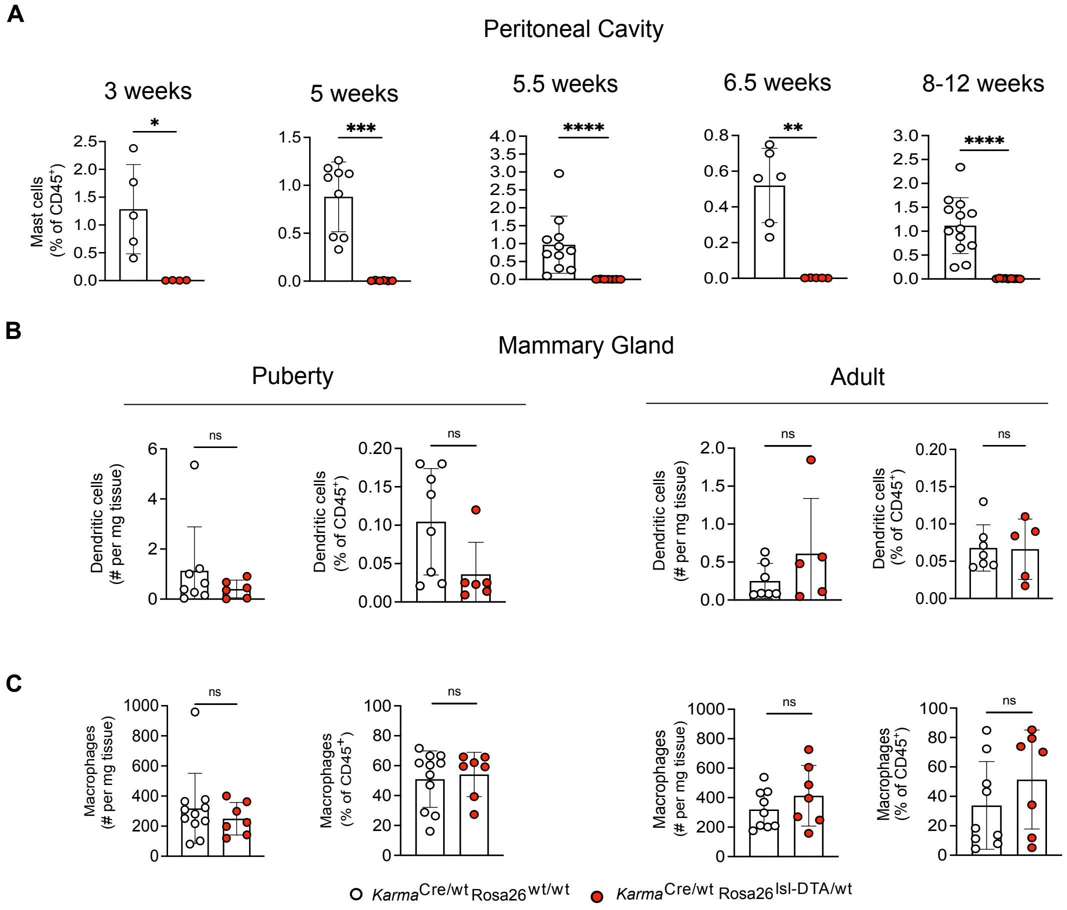

Figure S2 (related to Figure 2): Mast cells and dendritic cells in *Karma*^{Cre}:Rosa26$^{lsl\text{-}DTA}$ mice. (A) Mast cell numbers and/or relative abundance within hematopoietic compartment (% within CD45^{+} cells) of the peritoneal cavity of *Karma*$^{Cre/wt}$:Rosa26$^{lsl\text{-}DTA/wt}$ (red) compared to mast-cell proficient littermate *Karma*$^{Cre/wt}$:Rosa26$^{wt/wt}$ controls (white) at the indicated ages, as measured by flow cytometry. **(B,C)** Dendritic cell **(B)** and macrophage **(C)** numbers and relative abundance within hematopoietic compartment (% within CD45^{+} cells) and numbers in the mammary gland of *Karma*^{Cre}:Rosa26$^{lsl\text{-}DTA}$ mice at puberty (5.5 weeks; left) and adulthood (8-12 weeks; right). Data are cumulative of at least 4 individual mice per group from at least 2 or more independent experiments. Data are presented as mean with error bars indicating the SD. Data are shown as mean with SD as error bars. ****p<0.0001, ***p<0.001, **p<0.01, *p<0.05 as determined by Mann- Whitney test. ns = not significant.

Figure S3

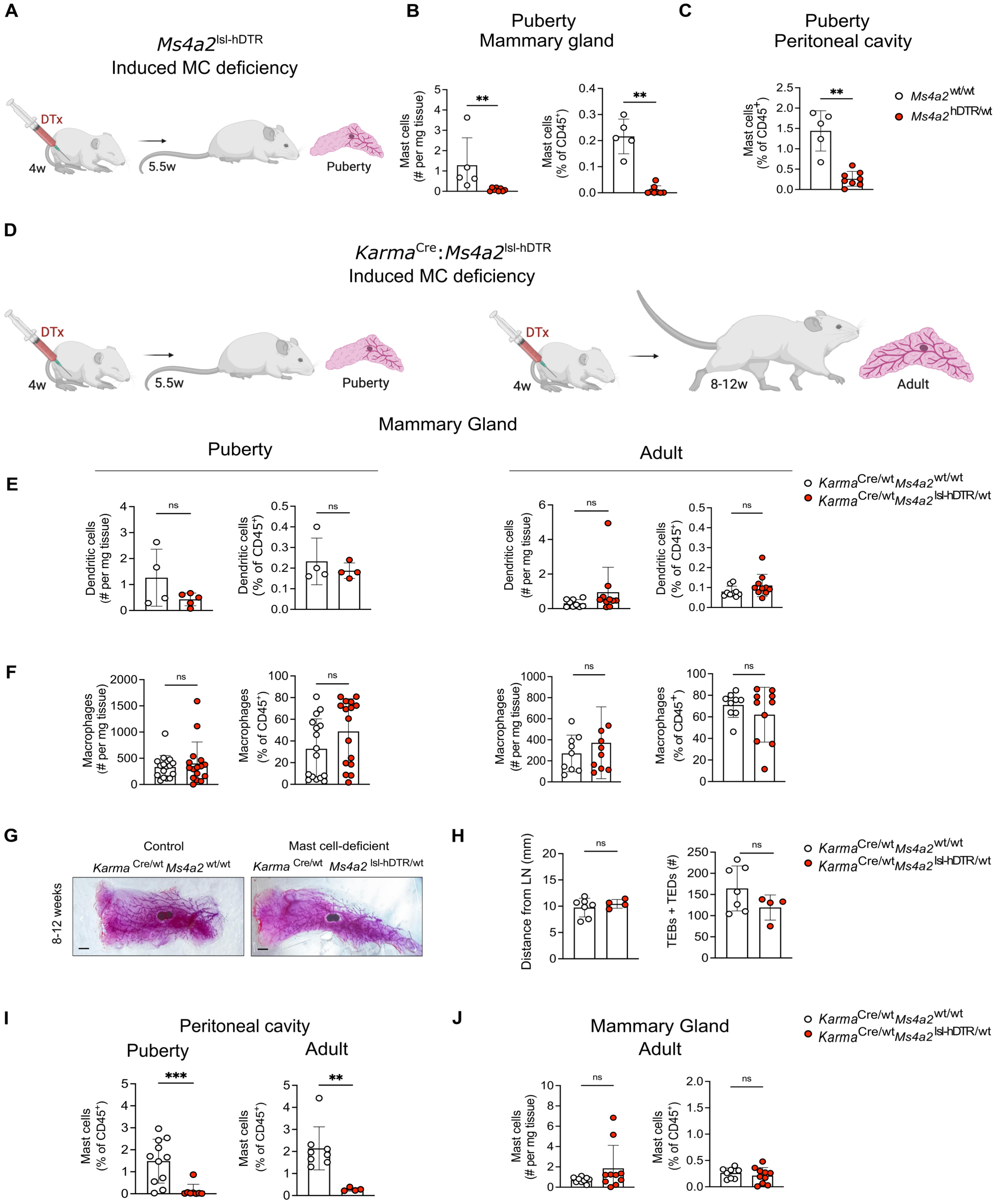

Figure S3 (related to Figure 3): Mast cells, dendritic cell and mammary gland branching in *Ms4a2*^{hDTR} and *Karma*^{Cre}:*Ms4a2*$^{lsl-hDTR}$ mice. To induce mast cell ablation, *Ms4a2*^{hDTR} (A-C) or *Karma*^{Cre}:*Ms4a2*$^{lsl-hDTR}$ mice (D-J) were treated with 1mg Diphtheria toxin at 4 weeks of age by subcutaneous injection into the mammary gland fat pad. Littermate control animals (*Ms4a2*$^{wt/wt}$ or *Karma*$^{Cre/wt}$:*Ms4a2*$^{wt/wt}$)

were treated the same way. **(A)** Scheme depicting the use of the *Ms4a2*^{hDTR} model with depletion at 4 weeks and analysis at puberty. **(B, C)** Flow cytometry was used to determine mast cell numbers and relative abundance within hematopoietic compartment (% within CD45^{+} cells) in the mammary gland **(B)** and peritoneal cavity **(C)** at puberty (5.5 weeks). **(D)** Diagram showing the use of the *Karma*^{Cre}:*Ms4a2*$^{lsl\text{-}hDTR}$ model with depletion at 4 weeks and analysis at either puberty or in adulthood. **(E)** Dendritic cell and **(F)** macrophage cell numbers and relative abundance within hematopoietic compartment (% within CD45^{+} cells) in the pubertal mammary gland of *Karma*^{Cre}:*Ms4a2*$^{lsl\text{-}hDTR}$ mice following treatment with Diphtheria toxin at 4 weeks. Data were obtained using flow cytometry. **(G-J)** Effects of Diphtheria toxin administration to 4-weeks-old *Karma*^{Cre}:*Ms4a2*$^{lsl\text{-}hDTR}$ mice, determined at puberty or in adulthood. **(G, H)** Branching as determined by Carmine staining of pubertal mammary glands from adult mast cell-deficient (*Karma*$^{Cre/wt}$:*Ms4a2*$^{lsl\text{-}hDTR/wt}$ mice; right/red) and mast cell-proficient mice (*Karma*$^{Cre/wt}$:*Ms4a2*$^{wt/wt}$ mice; left/white). **(G)** Representative images of mammary glands stained with Carmine. Scale bars = 1mm. **(H)** Quantification of (G). The extent of branching was measured as distance of branches and the number of terminal end buds (TEBs) and end ducts (TEDs) from the middle of the lymph node. **(I)** Effects of Diphtheria toxin treatment on the number of mast cells were assessed in the peritoneal cavity. Flow cytometry was used to determine mast cell numbers and relative abundance within hematopoietic compartment (% within CD45^{+} cells) of the peritoneal cavity during puberty (5.5 weeks; left) and adulthood (8-12 weeks; right). **(J)** Adult mammary gland mast cell numbers and relative abundance within hematopoietic compartment (% within CD45^{+} cells) assessed by flow cytometry. Data in (B, C, E, F, H, I, J) are cumulative of at least 4 individual mice per group from at least 2 or more independent experiments. Data in (B, C, E, F, H, I, J) are presented as mean with error bars indicating the SD. ***$p<0.001$, **$p<0.01$ as determined by Mann-Whitney test or unpaired t-test as applicable. ns = not significant.

Figure S4

A

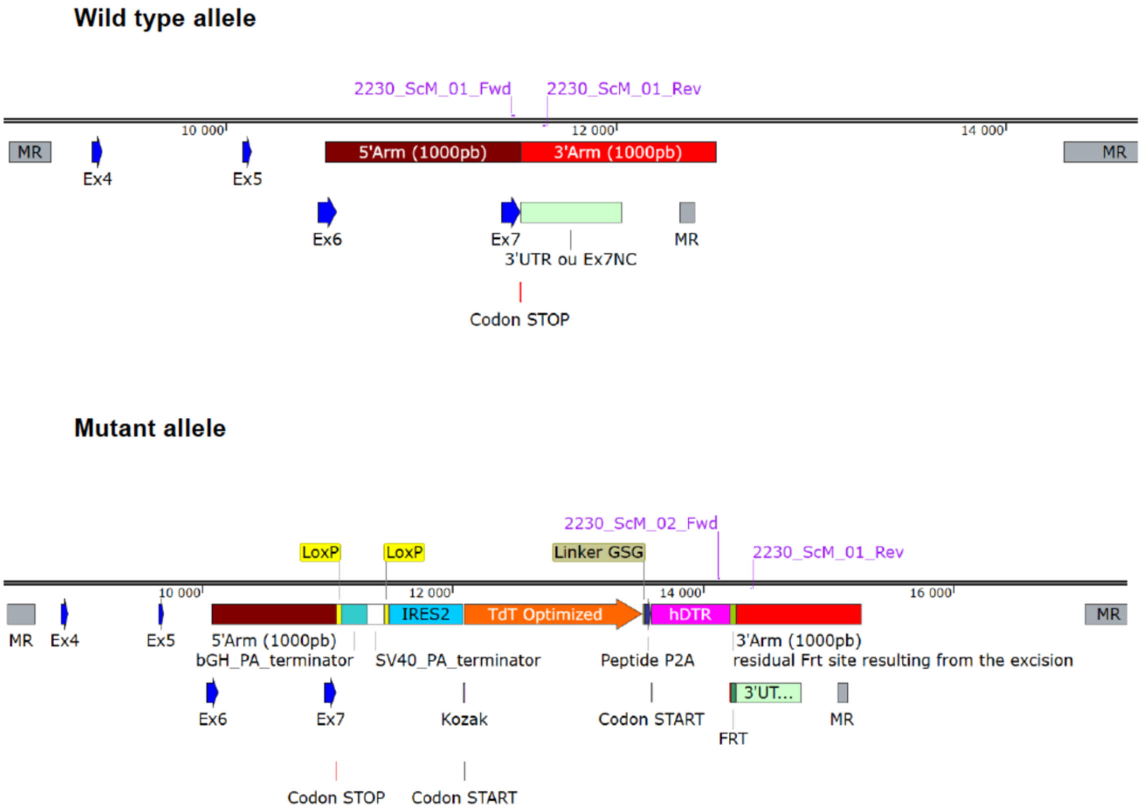

Possible genotypes:

Ms4a2$^{wt/wt}$

Ms4a2$^{lsl\text{-}DTR/wt}$

Ms4a2$^{lsl\text{-}DTR/lsl\text{-}hDTR}$ (not used in this study)

Figure S4 (related to Methods): Genetic modifications in *Ms4a2*$^{lsl\text{-}hDTR/wt}$ mice. Schematic overview of genetic alterations. A knock-in of the *Ms4a2* murine gene (Ensembl number ENSMUSG00000024680) was carried out by the insertion of an LSL_tdT_P2A_hDTR sequence in the 3'UTR of the *Ms4a2* gene. A residual FRT element is present just after the LSL_tdT_P2A_hDTR sequence in the mutant allele, a result of the deletion of the NeoR cassette. This NeoR cassette provided neomycin resistance as a positive marker during the selection of mutant mES cells. Ex: Exon, P2A: Peptide 2A, SV40: Simian Virus 40, hDTR: human Diphtheria toxin receptor. Possible genotypes are indicated.
